# Supplementary material for: Quantification of Serum Exosome Biomarkers Using 3D Nanoporous Gold and Spectrophotometry
Source: Sensors (Basel). 2022 Aug 24;22(17):6347. doi: 10.3390/s22176347 (PMC9460504; doi:10.3390/s22176347)
Supplement: Supplementary file 1 [file sensors-22-06347-s001.zip › sensors-1647859-supplementary.pdf]

Supplementary Materials

Quantification of Serum Exosome Biomarkers Using 3D Nanoporous Gold and Spectrophotometry

Amera Al Mannai [1], Tareq Al-Ansari [1] and Khaled M. Saoud [2,*]

1 College of Science and Engineering, Hamad Bin Khalifa University, P.O. Box 34111, Doha, Qatar; ameraalmannai@hbku.edu.qa (A.A.M.); talansari@hbku.edu.qa (T.A.-A.)

2 Liberal Arts and Science Program, Virginia Commonwealth University, P.O. Box 8095, Doha, Qatar

* Correspondence: s2kmsaou@vcu.edu

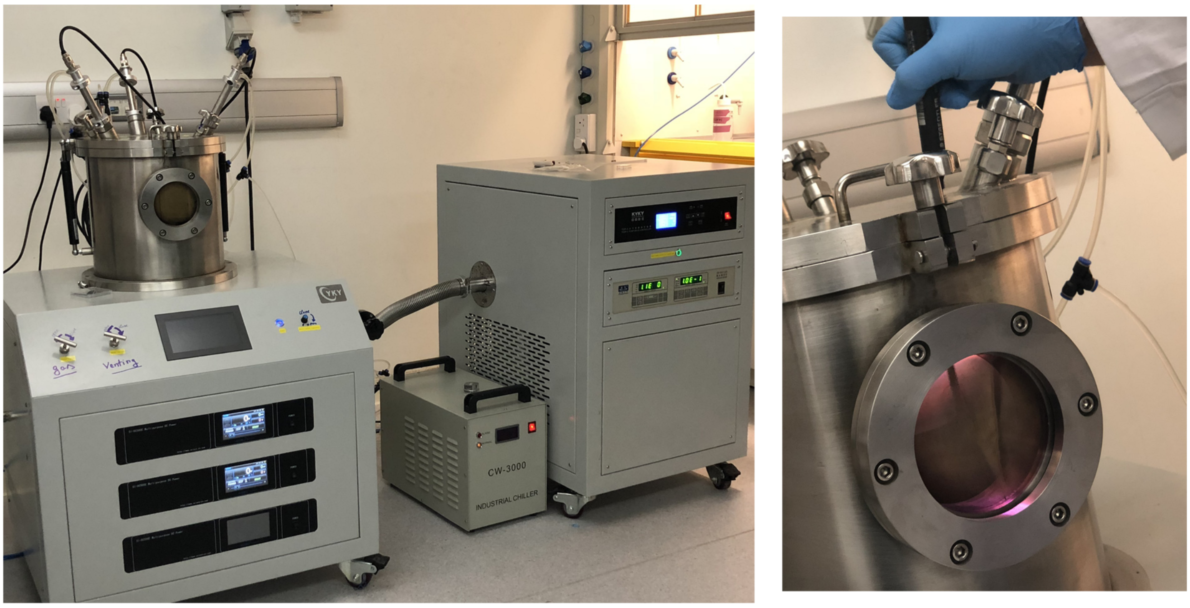

Figure S1. Sputtering Machine.

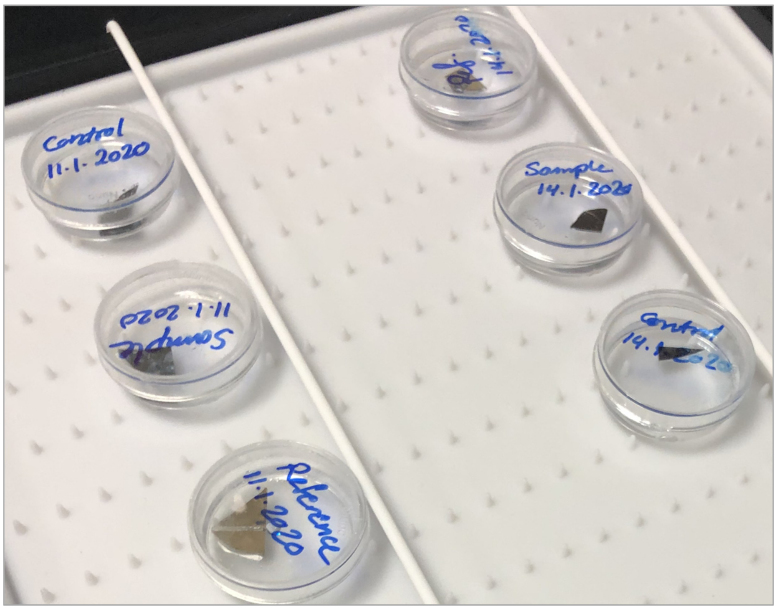

Figure S2. Porous, nonporous (Reference), and Porous gold substrates with different antibodies (Control).

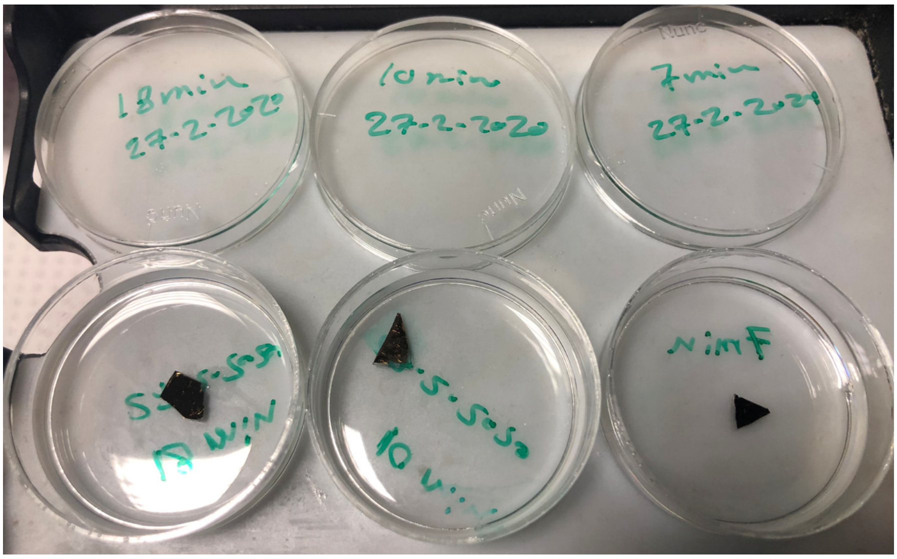

Figure S3. Porous gold substrates treated at different times.
